# Psychosocial Determinants of Behavioral Health in Latinx Americans Nationwide: A Systematic Review Highlighting Cultural Strength Factors

**DOI:** 10.3390/ijerph22111715

**Published:** 2025-11-13

**Authors:** Amy L. Ai, Zhe Yang, Michaé D. Cain, Thomas Knobel

**Affiliations:** 1 Institute of Longevity, Pepper Institute on Aging and Public Policy, Florida State University, Tallahassee, FL 32306, USA; 2 College of Social Work, Florida State University, Tallahassee, FL 32306, USA; zyang5@fsu.edu (Z.Y.); mdcain@fsu.edu (M.D.C.); 3 Independent Researcher, 557 Wall Road, Spring Lake, NJ 07762, USA; tak21@fsu.edu

**Keywords:** NLAAS, depression, anxiety, substance use, Latinx Americans, psychosocial determinants, behavioral health, risk and protective factors, trauma, acculturation, immigration

## Abstract

Objectives: Latinx Americans represent the largest ethnic minority group (nearly 19% of the U.S. population). Their behavioral health has received increasing attention as they exhibit elevated prevalence rates of anxiety (ANX), depression (DEP), and substance use disorders (SUDs). The National Latino and Asian American Study (NLAAS) is the first national population-based mental health study of Latinx Americans and is the most comprehensive resource for understanding their behavioral health. This systematic review aims to synthesize peer-reviewed publications using the NLAAS dataset to identify psychosocial determinants of the three key outcomes. Method: We followed PRISMA to search for English peer-reviewed articles published in EBSCO, Embase, PsycINFO, Web of Science, and PubMed. Inclusion criteria were as follows: (1) Latinx in the NLAAS database; (2) ANX, DEP, or SUD; (3) risk or protective factors; and (4) peer-reviewed publications in English. Search terms such as Latino, Latina, anxiety, depressive symptoms, substance abuse, and NLAAS were used to search for relevant articles. Two authors screened the articles independently and extracted data from each study. Results: Thirty-two studies published between 2007 and 2024 were included in our final review. Among them, 12 studies investigated ANX, and 17 studies examined DEP and SUD, respectively. Sixteen studies assessed protective factors. Ten articles assessed multiple key outcomes. All risk factors were grouped into three categories: Trauma and negative relationships (e.g., childhood maltreatment, negative family relationship, traumatic life experience), acculturation- and immigration-related factors (e.g., nativity, acculturation experience, English proficiency, discrimination), and sociodemographic and social participation factors (e.g., gender, education, income level). Protective factors such as family cohesion, religious activity, gender, and education were also identified. Conclusion: This first systematic review provided comprehensive NLAAS findings on multifaceted cultural, social, and intrapersonal factors that were either negatively or positively associated with three behavioral health outcomes within the U.S. Latinx population. Potential mechanisms by which risk and protective factors influence their mental health, as well as limitations of this review, were discussed. Findings of this review can inform culturally responsive prevention strategies and interventions to reduce behavioral health disparities and to improve mental health outcomes among Latinx Americans.

## 1. Introduction

With most of its members being foreign-born individuals, Latinx Americans constitute the fastest-growing ethnic group in the United States (U.S.), and their numbers are projected to more than double between 2000 and 2050 [1]. Currently, the Latinx population still represents the largest ethnic minority group in the U.S., comprising approximately 19% of the total population [2]. This demographic trend has drawn growing attention to the mental and behavioral health of the Latinx population, particularly in connection with the development of the National Latinx and Asian American Survey (NLAAS) [3,4]. This is the first national household representative survey on the overall behavioral and mental health issues of the Latinx American community, and also accounts for acculturation and immigration factors. The NLAAS database provides highly valuable information on both risk and protective factors related to the diagnostic criteria of Latinx Americans nationwide [3,4]. By following the widely accepted Diagnostic and Statistical Manual of Mental Disorders, Fourth Edition (DSM-IV) criteria [5], the NLAAS employs a standardized assessment framework for identifying key behavioral health conditions (e.g., anxiety, depression, and substance use disorders), ensuring the findings are comparable across studies and contribute to a more cohesive evidence base. Thus, the NLAAS dataset plays a key role in advancing multidisciplinary understanding of this U.S. subpopulation.

Depression (DEP), anxiety (ANX), and substance use disorder (SUD) are major public health challenges in the U.S. [6,7,8]. Existing research has shown the prevalence and the public health harm of DEP, ANX, and SUD in the general U.S. population [6,7,9,10,11]. In 2024, among individuals 18 years and older, 7.4% of the U.S. population had past-year ANX and 8.2% had past-year DEP. Among individuals 12 and older, approximately 17% had SUD [11]. Together, these conditions substantially contribute to health outcomes such as years lived with disability, premature mortality, and increased health care costs [9,10].

Currently, Latinx Americans report higher levels of ANX and DEP than the general population. Almost one-third of them have reported depressive symptoms, whereas the rate of SUD is slightly lower [11]. Estimates suggest that 9.9% adult Latinos in the U.S. have experienced mild ANX symptoms, 3.6% experienced moderate symptoms, and 2.6% experienced severe anxiety in the past two weeks [12]. In 2021, approximately 30% of the Latinx population aged 12 and older had past-year DEP, and 15.7% had past-year SUD [11]. Accordingly, Latinx individuals suffered from a considerably higher risk of ANX, DEP, and SUD than the general population in the U.S.

In public health, behavioral science, and social science, different disciplines have different conceptualizations of psychosocial determinants [13,14,15]. Psychosocial determinants are powerful factors that shape the pathways leading to illness, though they do not function as a direct cause of the disease [16]. For example, lower socio-economic status does not directly cause a disease, but it exerts a persistent influence through mechanisms such as chronic stress, food insecurity, transportation barriers, and/or hazardous housing conditions that increase vulnerability to adverse health outcomes (e.g., inadequate access to care, poorer health conditions) [17,18,19,20]. To date, no study has provided comprehensive information on psychosocial determinants derived from the aggregated NLAAS findings for DEP, ANX, and SUD among the Latinx American population.

This systematic review aims to reveal such determinants, especially risk and protective factors of three behavioral health conditions (DEP, ANX, and SUD) in the Latinx American population. Psychosocial determinants were extracted from peer-reviewed publications that investigated their relationships with DEP, ANX, and SUD using the NLAAS database. To inform researchers and clinical professionals (e.g., physicians, nurses, psychologists, social workers, and public health practitioners) in multidisciplinary fields, the extracted risk factors were categorized into three groups (i.e., trauma and negative relationships, acculturation- and immigration-related factors, and sociodemographic and social participation). We also extracted protective factors from the included articles that address a critical but underreported aspect of the literature to advance clinical assessments and interventions for the targeted population.

## 2. Method

### 2.1. Design

This systematic review was conducted based on the guidelines from the Preferred Reporting Items for Systematic Reviews and Meta-Analyses (PRISMA) [21]. Selected databases were used for searching peer-reviewed English articles that used data from the NLAAS dataset on risk and protective factors for ANX, DEP, and SUD among Latinx populations.

### 2.2. Material: Publications from the NLAAS Database

The NLAAS is the first national population-based mental health study of Latinx and Asian Americans, with data collected from May 2002 to December 2003 [3,4]. It has three major aims: (1) To estimate the 12-month and lifetime prevalence of psychiatric disorders and the rates of mental health service utilization; (2) to examine how social position, environmental context, and psychosocial factors are associated with psychiatric disorders and service use; and (3) to compare prevalence and service utilization patterns between Latinos and Asian Americans. Specifically, the NLAAS data included social demographics, mental health screening and diagnosis, health service usage, evaluations, acculturation factors, and other variables. The sampling procedure for the NLAAS was previously documented, and weights were developed to correct sampling bias in both the total sample and for Latinx American subgroups [3,4].

The NLAAS classified Latinx participants into four groups: Mexicans, Puerto Ricans, Cubans, and an “Other” category (e.g., Costa Ricans, Ecuadorans, Guatemalans, and Hondurans). The total sample size of the NLAAS was 4649, including 2554 Latinx Americans and 2095 Asian Americans, all aged 18 years or older and residing in the U.S. The NLAAS participants were interviewed by trained bilingual interviewers. For quality control, a random sample of participants who had completed interviews were recontacted to validate the data.

In addition, the NLAAS adapted criteria used in the World Health Organization Composite International Diagnostic Interview (WHO-CIDI) to capture mental health history over the previous 12 months [22]. The criteria in WHO-CIDI are compatible with those listed in DSM-IV. The World Mental Health Survey Initiative version of the WMH-CIDI was used to assess the prevalence of ANX, DEP, and SUD in NLAAS [23].

### 2.3. Search Strategy

A comprehensive literature search was conducted in July 2025 across five electronic databases: EBSCO, Embase, PsycINFO, Web of Science, and PubMed. The search strategy focused on searching articles that examined risk and protective factors for DEP, ANX, and SUD among U.S. Latinx populations using the NLAAS dataset. The search terms were constructed by combining three groups of keywords: (1) Terms referring to the target population, such as “Latino,” “Latina,” “Latinos,” “Latinas,” and “Latinx”; (2) terms describing the key outcomes, including ANX (e.g., “anxiety,” “anxiety disorder,” “generalized anxiety disorder”), DEP (e.g., “depressive disorder,” “depressive symptoms,” “major depressive disorder”), and SUD (e.g., “substance abuse,” “drug use,” “drug abuse,” “dependence,” “addiction”); and (3) terms related to the NLAAS database, such as “NLAAS” and “National Latino and Asian American Study”. To ensure the search was as inclusive as possible, no restriction was set on publication year, geographic location, or specific database fields.

### 2.4. Inclusion and Exclusion Criteria

Studies that met all the following criteria were included in this review: (1) Must have sampled and separately reported results for the Latinx population; (2) must have outcome variables including ANX, DEP or SUD; (3) must have results of risk or protective factors that relate to the three key outcomes; and (4) must be peer-reviewed empirical articles that have been published in English. We excluded articles that (1) studied the Latino population but reported results combining participants in other ethnic groups (e.g., Asian population); (2) used other outcomes (e.g., self-rated mental health, mental health service utilization); (3) used other databases that did not include NLAAS (e.g., National Survey of American Life); or (4) were not peer-reviewed empirical articles (e.g., reviews, abstracts, and dissertations).

### 2.5. Study Screening

All returned results were imported into the web-based systematic review software Covidence (Melbourne, Australia, https://www.covidence.org) to eliminate duplicate and irrelevant publications. Each identified article was screened by two authors (Z.Y. and M.C.) independently to determine eligibility via reviewing of titles and abstracts. Then, a full-text review was conducted for the remaining articles by the same two authors independently. Z.Y. and M.C. resolved discrepancies through meetings by presenting their rationale and evidence for inclusion or exclusion decisions. If consensus could not be reached, the senior author (A.A.) provided suggestions to make the final decision. The inter-rater reliability (IRR) coefficient for each phase was calculated by Covidence. IRR values were 0.67 for title and abstract screening and 0.72 for full-text review, indicating moderate to substantial agreement between reviewers [24].

### 2.6. Data Extraction

Data extraction primarily focused on study characteristics and principal findings. Study characteristics included author, publication year, sample size, age, and gender. Key findings included three targeted outcomes, risk and protective factors, and consistency of evidence.

## 3. Results

### 3.1. Searching and Screening Process

**Figure 1:**
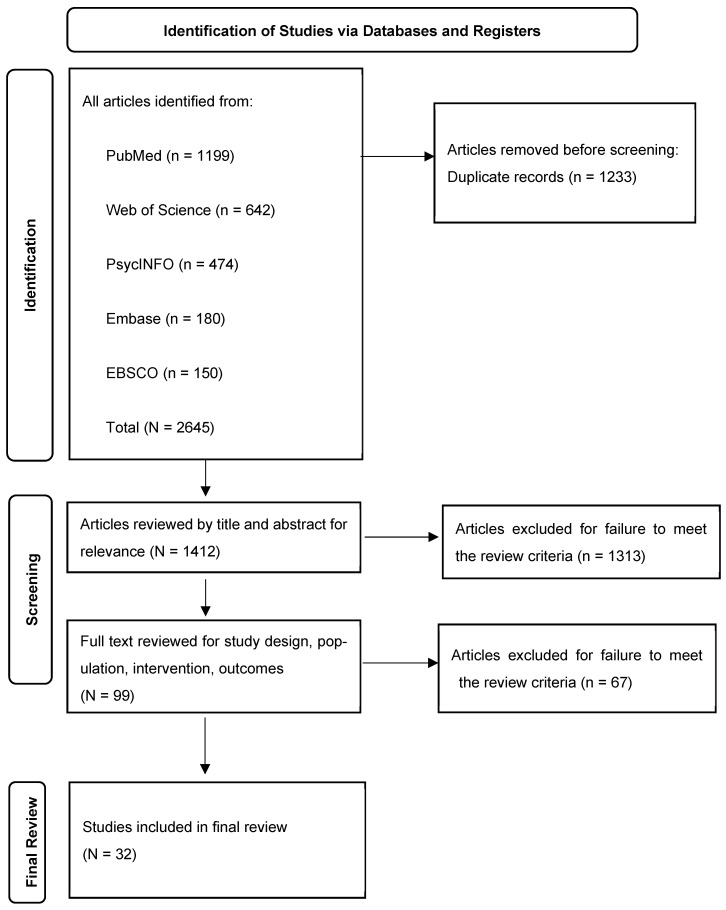
PRISMA flowchart of process for systematic review and identification.

The initial search process yielded 2645 articles, with 1199 of the results from PubMed, 642 from Web of Science, 474 from PsycINFO, 180 from Embase, and 150 from EBSCO. Covidence identified and removed 1232 duplicated studies, and 1 article was manually identified as a duplicate. Full search and results are shown in Figure 1. Thus, 1412 articles were screened using titles and abstracts, and 1313 articles were excluded, leaving 99 articles for full-text review. From this total, 67 studies were excluded because they were not empirical studies (e.g., review, dissertation, abstract) (*n* = 19); did not have outcomes of ANX or DEP or SUD (*n* = 14); did not separately report ANX or DEP or SUD (*n* = 10); did not separately report results for the Latino American population (*n* = 7); did not include risk or protective factors (e.g., studies that report prevalence or group difference) (*n* = 12); and either ANX or DEP or SUD was the independent variable rather than the outcome (*n* = 5). Thirty-two studies were included in the final sample for review.

Among the 32 included articles, 12 studies investigated ANX, 17 studies examined DEP, and 17 studies used SUD as the outcome. Ten articles assessed multiple key outcomes. Regarding psychosocial determinants, 29 studies identified risk factors, and 16 studies identified protective factors. Only thirteen studies reported both risk and protective factors.

Among the 12 studies examining ANX, 10 identified risk factors and 7 reported protective factors. Of the 17 studies on DEP, 14 identified risk factors and 10 reported protective factors. Similarly, for the 17 studies focusing on SUD, 15 identified risk factors and 9 reported protective factors. Overall, the results on risk and protective factors were largely consistent across all studies. Only six factors (i.e., social support, religious activities, family conflict, discrimination, education, and income level) yielded mixed findings in five studies [25,26,27,28,29].

### 3.2. Study Characteristics

All studies were published in English from 2007 to 2024 (Table 1). More than half of the studies (*n* = 20) focused on the general Latinx population, four examined Latinx immigrants, and three examined Latinas only (Latin American women). Only one study focused on each of the following groups: Latinos (Latin American men) (*n* = 1), Hispanic adults who immigrated during their youth (*n* = 1), Mexican populations (*n* = 1), older Latinx adults (*n* = 1) and Hispanics (*n* = 1). The range of the sample size for these 32 included studies was 231 to 3264 participants. The average number of participants was 2161 per study (SD = 764.86). All participants were aged between 18 and 97 years old, with 29,988 females (54.50%). Five studies did not report gender data [30,31,32,33,34].

Risk factors for ANX, DEP, and SUD are separately presented in the following sections. Given the limited evidence, all protective factors were integrated and reported in the final section.

### 3.3. Risk Factors for Anxiety (Table 2)

#### 3.3.1. Trauma and Negative Relationships

Various types of traumatic experiences have been reported to be associated with ANX among Latinx Americans. Among Hispanic women, a history of maltreatment was significantly related to anxiety disorder [56]. Specifically, witnessing violence during childhood was associated with significantly higher odds of developing an anxiety disorder. The likelihood of anxiety disorders was about four times greater for women who had been beaten during childhood and who experienced sexual abuse compared to their counterparts without such experiences [56]. Another study also found that each one-unit increase in traumatic experiences related to the 9/11 attacks was linked with a 9% higher likelihood of reporting anxiety disorders [44].

**Table 2:** Risk factors associated with ANX.

Categories	Factors	Number of Studies	Effect Size Range	References
Trauma and Negative Relationships	Maltreatment (witness violence, beaten, sexual assault)	1	1.14–4.71	[56]
	Negative life experiences (e.g., 9/11 terrorist attacks)	1	1.021–1.204	[44]
	Negative family interactions	4	1.016–2.215	[29,32,44,56]
Acculturation- and Immigration-Related Factors	English proficiency	1	1.096–2.790	[54]
	Acculturation experience	1	1.15–2.89	[46]
	Spoke both English and Spanish while growing up	1	1.026–2.668	[54]
	Discrimination	3	1.028–2.28	[34,44,47]
Sociodemographic and Social Participation Factors	Age	1	1.010–1.055	[29]
	Infrequent religious attendance	1	1.17–4.73	[33]
	Out of labor force	1	1.172–3.919	[54]
	Marital status	1	1.096–2.636	[54]
	Gender (female)	1	1.42–2.24	[55]

Experiences of negative relationships have also emerged as an important risk factor for ANX among Latinx populations. For example, negative family interactions, such as family discord, were significantly related to all anxiety disorders except panic disorder [32]. Family cultural conflict was linked to higher odds of anxiety disorders among Latinx Americans overall [44], and Hispanic women in the United States [56]. In addition, each unit increase in negative family interactions was associated with more than 50% higher likelihood of generalized anxiety disorder among Latinas [29]. A significant interaction was also observed between witnessing violence and family cultural conflict among Hispanic women, which was also associated with anxiety disorders [56].

#### 3.3.2. Acculturation- and Immigration-Related Factors

English proficiency is a critical indicator of adaptation during acculturation and has also been associated with ANX. Research has shown that Latinx individuals with high English language proficiency and those who grew up speaking both Spanish and English exhibited a greater likelihood of experiencing anxiety or social anxiety disorders [39,54].

Acculturation experiences have been shown to play a key role in the development of anxiety among the targeted population. One study observed that high levels of acculturative stress were linked to reporting lifetime anxiety among Latino immigrants [46]. Also, third-generation Latinos (U.S.-born, with both parents also U.S.-born) were found to have increased odds of ANX [55].

Discrimination represents another key acculturation-related stressor linked to ANX. The experiences of discrimination were associated with increased odds of having an anxiety disorder [44]. A relatively high frequency of discrimination was significantly linked to a diagnosis of mood or anxiety disorders [47]. Additionally, specific forms of discrimination, such as post-9/11 discrimination, were associated with a 39% higher likelihood of reporting an anxiety disorder [34].

#### 3.3.3. Sociodemographic and Social Participation Factors

Sociodemographic factors and social participation have also been linked to anxiety among Latinx populations. One study found that each additional year of age was associated with an approximately 2% higher likelihood of being diagnosed with generalized anxiety disorder [29]. Gender differences were also observed, as Latinas had significantly higher odds of mood or anxiety disorders compared to Latinx men [47,55]. Also, Latinx individuals who were out of the labor force and widowed, divorced, or separated, were more likely to experience social anxiety disorder [54].

In terms of social participation, infrequent religious attendance (less than once per year) was associated with higher rates of anxiety disorders among Hispanics [33].

Overall, the findings on risk factors for ANX were generally consistent across the three main categories.

### 3.4. Risk Factors for Depression (Table 3)

#### 3.4.1. Trauma and Negative Relationships

Concerning traumatic experiences, Hispanic women who had witnessed violence during childhood had significantly higher odds of developing a depressive disorder compared to those who had not [56].

As for negative family interactions, family conflict or family cultural conflict was significantly associated with increased risk for DEP or major depressive disorder (MDD) among Latinx populations [25,44,48,56]. In addition, visits back home were associated with increased odds of experiencing MDD, with the association being stronger among women than men [37]. Potential explanations include caregiving demands and social stress arising from strained relationships with family members (e.g., kin, spouses, or children left behind) during these visits [37]. Also, traumatic experiences related to the 9/11 terrorist attacks were associated with an 8% increase in the odds of reporting a depressive disorder among Latinx [44].

**Table 3:** Risk factors associated with DEP.

Categories	Factors	Number of Studies	Effect Size Range	References
Trauma and Negative Relationships	Maltreatment (witness violence)	1	1.13–2.87	[56]
	Family conflict or family cultural conflict	1	β_direct = 0.24	[48]
		2	1.09–1.323	[44,56]
		1	*0.16–4.87*	[25]
	Visits back home	1	1.01–1.06	[37]
	Traumatic experience (e.g., 9/11)	1	1.029–1.134	[44]
Acculturation- and Immigration-Related Factors	U.S.-Born	1	1.144–18.81	[29]
	Third-generation	1	1.08–2.01	[55]
	Acculturative stress and dissonant acculturation	2	β _(Acculturation stress)__direct = 0.094; β _(Dissonant acculturation)__direct = 0.18; Acculturative stress: 1.40–3.28	[43,46]
	Discrimination	1	β_direct = 0.15;	[48]
		1	1.044–1.096	[44]
		1	*0.13–3.83*	[25]
	Having resided longer than 5 years in the United States	1	1.20–5.31	[36]
Sociodemographic and Social Participation Factors	Gender (female)	4	1.24–3.36; β_direct = 1.085	[36,43,45,55]
	Religious attendance	1	1.17–4.73	[33]
	Ethnicity	1	1.15–2.75	[31]
	Marital status	2	1.20–2.19	[45,55]
	Being out of the labor force	1	1.36–4.68	[42]
	Lower social economic status (3)	3	1.01–8.20 β_direct = −0.123	[31,36,43]

Note. Italics indicate findings with mixed effects. β represents standardized path coefficients.

#### 3.4.2. Acculturation- and Immigration-Related Factors

Regarding acculturation experiences, Latinx Americans with excellent or good English language proficiency were more likely to have higher rates of depressive disorders compared to those who had less proficiency [39].

As for immigration experiences, compared to Latina immigrants who had been in the United States for fewer than 5 years, U.S.-born respondents were five times more likely to have a diagnosis of MDD [29,31,36]. Furthermore, third-generation Latinos (U.S.-born, with both parents also U.S.-born) were found to have increased odds of a depressive disorder [55].

Acculturative stress and dissonant acculturation both had direct impacts on depressive symptoms among Hispanic adults who immigrated as youth [43]. For Latinx immigrants, high levels of acculturative stress were associated with the presence of a depressive disorder within the past 12 months [46,48]. Discrimination is also a significant risk factor during the acculturation process. Higher levels of discrimination have been shown to increase the risk of DEP among Latinx populations [25,44,48].

#### 3.4.3. Sociodemographic and Social Participation Factors

Quite a few NLAAS studies also found that Latina Americans who immigrated during their youth, as well as Latinas in general, were more likely to experience the onset of MDD [31,36,43,45,55]. In terms of ethnicity, Latinx individuals were more likely to experience higher odds of DEP compared to individuals of Asian ethnicity [31]. Also, younger and unmarried/previously married Latinx individuals had a higher likelihood of experiencing MDD compared to their older and married counterparts [45,55].

Research also documented perceived social status as an important factor that influenced Latinx Americans’ mental health. Latinxs with lower subjective social status, as well as Hispanic adults who immigrated during youth who perceived downward social mobility or experienced a drop of three or more steps on the subjective social status (SSS) ladder, were more likely to report the diagnosis of major depressive episode (MDE) [31,36,43]. Not surprisingly, being out of the labor force was also related to higher odds of MDD [42].

Overall, most risk factors related to DEP were consistent across three main categories. However, the odds ratios for family conflict and discrimination reported by the same article had confidence intervals that included 1, suggesting mixed effects [25]. Since these two inconsistencies were reported in only one study, the isolated findings did not substantially alter the conclusion that family conflict and discrimination are risk factors for DEP.

### 3.5. Risk Factors for Substance Use Disorders (Table 4)

#### 3.5.1. Trauma and Negative Relationships

For traumatic experiences, childhood physical abuse or victimization was positively associated with lifetime SUD among Latino American men [27] and women [28]. It was revealed in a recent article that PTSD was a significant mediator in this relationship [35].

**Table 4:** Risk factors associated with SUD.

Categories	Factors	Number of Studies	Effect Size Range	References
Trauma and Negative Relationships	Childhood physical abuse or victimization	3	OR: 1.08–4.87 β_indirect = 0.06	[27,28,35]
	Problematic family relationships (man)	1	SMD: −0.56, −0.14	[40]
	More frequent interaction with friends (woman)	1	SMD: 0.22–0.71	[40]
	Family conflict	1	*0.20–1.99*	[25]
	Low engagement with one’s social network	1	1.73–9.29	[46]
Acculturation- and Immigration-Related Factors	English proficiency	5	1.03–47.83	[27,28,35,39,40]
	U.S. nativity	5	1.02–10.45	[27,28,35,39,41]
	Number of return visits to country of origin	1	1.00–1.07	[38]
	Second or third generational status	2	1.70–43.58	[39,55]
	Discrimination	1	β_direct = 0.12;	[48]
		6	1.01–7.02	[27,28,30,35,51,52]
		2	*0.23–1.0*	[25,26]
	High levels of racial/ethnic identity	1	NR	[26]
	Unfair treatment (1)	1	1.09–17.28	[51]
Sociodemographic and Social Participation Factors	Gender (male)	1	1.81–27.03	[30]
	Age	1	1.01–1.04	[27]
	interaction term (US. Born × lowest SSS)	1	1.67–6.65	[41]
	Religious coping	2	*Often [1.27–8.97]*	[27,28]
		1	Rarely [1.86–10.45]	[28]
	Social support	1	*1.32–3.84*	[28]
	Education (less educated)	1	1.03–3.45	[30]

Note. NR: Not reported. Italics indicate findings with mixed effects. β represents standardized path coefficients. SMD means Standardized Mean Difference.

Regarding negative relationships, SUD was also positively associated with problematic family relationships for Latino American men and was linked with more frequent interactions with friends for women [40]. Family conflict significantly predicted an elevated risk of smoking in the general Latino population [48]. Also, low engagement with one’s social network concurred with increased risk of lifetime substance-related disorders among Latinx immigrants [46].

#### 3.5.2. Acculturation- and Immigration-Related Factors

In relation to acculturation experiences, SUD was more likely to occur in Latinx individuals with higher English proficiency, including Latino American men [27], women [28], and the overall Latinx population [35,39]. Also, Latinx people who spoke English more frequently with family members had higher rates of SUD than those who spoke Spanish [40].

In terms of immigration experience, U.S. nativity was associated with alcohol use disorder (AUD) among Latinx. Individuals born in the U.S. had higher odds compared with those who were foreign-born [41]. Being U.S.-born was also positively linked with SUD among Latino American men [27,35], women [28], and the overall Latinx population [39]. Also, the risk of lifetime SUDs was higher among second-generation respondents (U.S.-born, with at least one foreign-born parent) and third-generation respondents (U.S.-born, with U.S.-born parents) compared to first-generation respondents (foreign-born) [39,55]. For Latino immigrants, the number of return visits to the country of origin in the past year was positively associated with current smoking status [38].

In addition, perceived discrimination was shown to be associated with SUD among Latino American men [27], women [28], Latinx nationwide [35], and particularly among Latinos with lower educational levels [30]. Higher levels of discrimination were linked with an increased risk of smoking and AUD among Latinx populations [25,26,48]. Low, moderate, and high levels of racial/ethnic discrimination were also associated with lifetime SUD, compared with individuals who reported no perceived racial/ethnic discrimination [51]. Furthermore, discrimination was significantly associated with an increased risk of AUD for Latino American women and an increased risk of drug abuse for Latino American men [52]. Finally, Latino men with high levels of racial/ethnic identity reported the greatest risk of being current smokers, compared to those with low levels of racial/ethnic identity [26]. Unfair treatment (i.e., everyday experiences perceived as unfair) was also associated with an increased risk of lifetime SUD [51].

#### 3.5.3. Sociodemographic and Social Participation Factors

Among Latinx men, older age was associated with higher levels of SUD [27], and a current SUD status was more prevalent in men than in women [30].

Among U.S.-born Latinos, those with the lowest SSS had the highest probability of reporting AUD, with the probability decreasing as SSS increased [41]. Also, the interaction term (U.S. SSS × college degree) indicated that college-educated Latinos at lower U.S. SSS levels faced greater AUD risks [41].

Overall, most risk factors related to SUD were consistent across three categories. The odds ratios for family conflict and discrimination had confidence intervals that included 1, suggesting mixed effects [25,26]. As the impact of family conflict was reported in one of the two studies, additional evidence is needed to confirm its effects. For discrimination, mixed findings in two of nine studies are insufficient to change the general conclusion that it is a risk factor for SUD [27,28,30,35,48,51,52]. In addition, religious coping and social support showed risk effects on SUD, which were inconsistent with findings from other studies [27,28]. Detailed elaboration is provided in the Section 4.4.

### 3.6. Protective Factors (Table 5, Table 6 and Table 7)

#### 3.6.1. Positive Relationships

Five studies have shown that greater family cohesion was associated with substantially lower risks of mental disorders, including generalized anxiety disorder, panic disorder, and both 12-month and lifetime depressive disorders among Latino immigrants and older Latino adults [29,32,46,53]. In addition, the absence or infrequency of family conflict was linked with lower risk of anxiety and substance-related disorders, underscoring the protective role of a harmonious family environment [46].

**Table 5:** Protective factors associated with ANX.

Categories	Factors	Number of Studies	Effect Size Range	References
Trauma and Negative Relationships	Family cohesion	3	0.28–0.999	[29,32,46]
Acculturation- and Immigration-Related Factors	Non-U.S.-born (1)	1	0.451–0.857	[54]
	Positive acculturative experiences	1	NR	[55]
	Spanish proficiency	1	0.425–0.985	[54]
Sociodemographic and Social Participation Factors	Religious attendance	1	β = −0.09	[50]
	Age	1	0.034–0.296	[54]
	Education (college degree or more)	1	0.41–0.87	[55]
	Gender (male)	1	0.42–0.73	[47]

Note. NR: Not reported.

**Table 6:** Protective factors associated with DEP.

Categories	Factors	Number of Studies	Effect Size Range	References
Trauma and Negative Relationships	Family cohesion	1	0.51–0.87	[53]
	Lower levels of family cohesion	1	0.01–0.70	[46]
Acculturation- and Immigration-Related Factors	Positive acculturative experiences	1	NR	[55]
Sociodemographic and Social Participation Factors	Education (>=13 yrs)	1	0.45–0.93	[55]
		1	*0.499–1.791*	[29]
	Age	1	0.94–0.96	[45]
	Remittances burden	1	0.67–0.98	[37]
	Gender (male)	1	0.41–0.78	[31]
	Religious attendance	2	β = −0.11 0.191–0.807	[44,50]
	Household income (middle income group)	1	0.24–0.84	[36]
	Having resided for less than 5 years in the United States	1	0.24–0.85	[31]
	Not a U.S. citizen	1	0.34–0.84	[31]

Note. NR: Not reported. Italics indicate findings with mixed effects. β represents standardized path coefficients.

**Table 7:** Protective factors associated with SUD.

Categories	Factors	Number of Studies	Effect Size Range	References
Trauma and Negative Relationships	Low level of, hardly ever, or have never had family conflict	1	0.25–0.92	[46]
Acculturation- and Immigration-Related Factors	Positive acculturative experiences	1	NR	[55]
Sociodemographic and Social Participation Factors	University education	3	0.19–0.98 β_direct = −0.29	[27,35,55]
	Gender (male)	1	0.41–0.78	[31]
	Religious attendance	2	0.07–0.87. β = −0.16	[28,50]
	Living in neighborhoods with higher concentration of Latinos/immigrants	1	0.80–1.98	[49]
	Sex (female)	3	β_direct = −1.72. 0.06–0.54	[26,35,55]
	Lower–middle household income	1	0.26–0.98	[46]

Note. NR: Not reported. β represents standardized path coefficients.

#### 3.6.2. Acculturation- and Immigration-Related Positive Factors

Immigration-related factors served as protective factors, including older age at immigration, shorter duration in the U.S., non-citizenship status, and Spanish proficiency. Latinx individuals who migrated after age 21, had lived in the U.S. for less than 5 years, were not U.S. citizens, and who had good/excellent Spanish proficiency were less likely to experience comorbid social anxiety disorder, drug abuse, and MDD [31,54]. Positive acculturative experiences serve as a comprehensive protective factor, as Latinxs who reported more favorable acculturation consistently had the lowest proportion across ANX, DEP, and SUD categories [55]. Economic responsibility through remittance practices such as sending money to family members, further appears to offer protection, with the burden associated with decreased odds of a major depressive episode [37].

#### 3.6.3. Sociodemographic and Social Participation Factors

Age acted as a protective factor among Latinx individuals, as one study found that those over 65 years old had a significantly lower likelihood of experiencing social anxiety disorder compared to people aged 18–34 [54]. Results of another study also found that each one-year increase in age was associated with about a 5% decrease in the odds of depression [45]. Gender also appeared to function as a protective factor, with females being less likely than males to experience lifetime SUD, specifically smoking and AUD [26,35,55], whereas men were less likely to experience mood/anxiety disorder and MDD [31,47].

Education serves as a strong sociodemographic protective factor. Latinas who had at least a high school diploma showed significantly lower odds of MDD [55]. Latino men, as well as the overall Latinx population who had a university degree reported reduced rates of SUD [27,29,35]. Interestingly, only individuals with middle household income (USD 35,000–USD 74,999) or lower–middle income exhibited significantly lower odds of MDD and SUD than their counterparts in the lowest- and high-income groups [36,46].

Social obligations and peer networks also help with lowering health risks. For example, remittance behavior has been associated with decreased likelihood of smoking among Latina immigrants, while friendship support was found to reduce smoking among Latino men [38]. Religious participation consistently acts as a protective factor, since frequent attendance at religious services was negatively related to anxiety, depressive disorders, and lifetime SUD among Mexican and the broader Latinx population [28,44,50]. In addition, living in neighborhoods with higher concentrations of Latinx immigrants resulted in a lower risk for past-year AUD compared to non-Latinx Whites [26].

The findings regarding protective factors for ANX, DEP, and SUD were generally consistent across the three main categories. However, the odds ratios for education included 1, suggesting the protective effects for education on DEP may not be completely consistent [29].

Taken together, the findings from this systematic review indicate that for ANX, negative family interactions (n = 4) and discrimination (n = 3) were the most consistently supported risk factors, whereas family cohesion (n = 3) served as the key protective factor. For DEP, gender (female; n = 4), family conflict (n = 3), and discrimination (n = 2) were the most consistently reported risk factors, with family cohesion or lower levels of family conflict (n = 2) and religious attendance (n = 2) acting as the main protective factors. For SUD, the most consistently supported risk factors were discrimination (n = 7), U.S.-born (n = 5), and higher English proficiency (n = 5), while education (n = 3), female (n = 3), and religious attendance (n = 2) emerged as the most consistent protective factors.

## 4. Discussion

To the best of our knowledge, this is the first systematic review using the aggregated data from peer-reviewed academic publications using the NLAAS dataset on psychosocial determinants of three prevalent mental health diagnoses (ANX, DEP, and SUD). Findings from this review offer a representative and comprehensive synthesis that captured key psychosocial determinants affecting three common mental health outcomes among this rapidly growing U.S. subpopulation. Given the collective cultural tradition of Latinx communities, the second category of risk factors (i.e., acculturation- and immigration-related factors) appears to be uniquely influential on behavioral health practices among the largest U.S. minority group. The other two categories (i.e., trauma and negative relationships, sociodemographic and social participation) are more universal across all U.S. subpopulations.

It is important to note that as the included studies utilized the NLAAS dataset, which is cross-sectional in nature, the temporal order between these factors and the outcomes could not be established. Thus, causal relationships cannot be inferred from these findings. The associations reported between the risk and protective factors and the three main outcomes were derived from correlational or regression analyses. Therefore, the results should be interpreted as indicative of associations rather than evidence of causation.

Our aggregated results do not serve as simple confirmations of past findings from the NLAAS publications over the past 20 years. In advancing the literature, the study highlights the limited evidence on underreported protective factors from the NLAAS database on their major mental health issues. Accordingly, we elaborate on these psychosocial determinants across the three mental health conditions.

### 4.1. Trauma and Negative Relationships

Collectivism is prominent in non-Western cultures, including those with Latinx roots (e.g., Latin America), where the collective “we” is viewed as more important than the individual “self” [57,58]. Within such communities, members’ personal goals and well-being are, in general, subsumed under those of the collective group [59]. Thus, interpersonal trauma and problematic family relationships could be essentially harmful to behavioral health. Only a few studies, however, have investigated and then supported the prediction of childhood mistreatment or early violent experiences for adulthood ANX, DEP, and SUD in the U.S. Latinx population, especially the Latina subpopulation in the NLAAS [27,28,35,56]. Given the limited number of studies, evidence from the NLAAS database have highlighted the importance and urgent need for more research and interventions on risk factors for this U.S. minority population.

Child maltreatment (CM)/victimization is an established major public health risk in developed countries, including the U.S. [60]. An estimated 17% and 8.3% of children experienced physical and sexual CM, respectively [61]. The prevalence of CM in Latinx children (11.0%) slightly exceeds that of White children (10.5%) [62]. Accordingly, the rate and the lifetime damage of CM on behavioral health are not unique for Latinx Americans. In 2024, Ai and colleagues revealed that it was physical abuse, and not sexual abuse, that causes more damage in the Latinx population, whereas sexual abuse appeared to be more harmful in the U.S. general population [35]. The differences may lie in the cultural roots between the two types of traditions. In addition, these authors found that only Latinx individuals who were traumatized by this early experience would develop SUD.

Not surprisingly, quite a few NLAAS publications present the negative impact of problematic social relationships, especially negative family interactions, on ANX, DEP, and SUD [25,29,32,40,44,48,54]. As the Latinx population is, on average, younger than the general population and most are foreign-born, immigration could induce generational conflicts between the traditional collectivist culture in many families and the individualistic values of most countries [63,64]. Family conflict and burden have been found to predict risk of mood disorders among Latinos in the NLAAS [39]. In particular, the odds of anxiety disorder were found to increase along with family discord and family cultural conflict, especially in Latinas [29,32,44,56]. Such strained relationships and problematic interactions were also associated with depressive disorders [25,44,48] and SUD [40], as well as smoking [46,48].

The literature has demonstrated the importance of harmonic family relationships for the mental health of general populations [29,32,46,53]. In Latinx communities that are predominantly shaped by Catholic and collectivist cultural traditions, members are expected to be more attentive to the needs of their family members than to their own [29,65]. Exposure to the individualistic American culture was found to increase Latinas’ likelihood of experiencing mental health problems because of cross-cultural conflicts and related discrimination, acculturation processes, and poverty [66]. Lorenzo-Blanco and Cortina revealed that acculturated Latinx had a pattern of family conflict, a lack of shared family values and cohesion, and frequently perceived discrimination [48]. The impact of family issues on their mental health should be understood and addressed within this inter-cultural context [29,32,56,67,68].

### 4.2. Acculturation- and Immigration-Related Factors

As noted, social relationship-related problems of Latinx Americans are closely intertwined with acculturation- and immigration-related factors [29,44,56,67,68]. Many studies revealed that the negative impact on Latinx mental health was influenced by the extent to which they were acculturated with the mainstream culture. These involve measures of (a) excellent English language proficiency [27,28,39]; (b) being U.S.-born [27,28,29,41]; (c) living longer in the United States [31]; and (d) being third-generation Latinxs [39,55]. The consequences involve all three conditions: ANX [39,46], DEP [29,31,39,41,43,45,54,55], and SUD [27,28,29,39,40,41].

Other important acculturation- and immigration-related factors include, but were not limited to (e) acculturative stress [43,44,46,48]; (f) immigrating as a teenager or at a younger age, rather than during adulthood, e.g., after age 21 [31,43,45,54]; (g) perceived downward social mobility after immigration [31,36,43], leading to a greater likelihood of experiencing DEP and ANX [54]; (h) more frequent re-visits to the country of origin [38]; and (i) perceived unfair treatment [51] leading to greater odds of SUD. As mentioned, the rate of depression is remarkably higher in the Latinx population than in the general U.S. population [11].

Particularly worth noting is the detrimental function of perceived discrimination, including post-9/11 discrimination, which was associated with anxiety [34,44,47] and SUD in numerous studies [25,26,27,28,30,35,48,51,52]. An undergirding rationale may lie in the *self-medication hypothesis* [69,70] and the *maladaptive coping assumption* [71,72]. Both theories posit that psychological symptoms (e.g., depression, anxiety) or painful emotional states (e.g., perceived unfair treatment) may precede the development of SUD. In other words, the aggregated results suggest that Latinx who were stressed out during their mingling with the mainstream culture may be more likely to use substances as a negative coping strategy. The consequences are harmful for both their well-beings and that of public health. Thus, the paramount evidence of the above risk factors from the NLAAS publications deserve professional attention regarding their effects on this ever-growing U.S. minority population.

Existing research has found that low self-reported rates of SUD in immigrant Latinx could also be associated with underutilization of specialized treatment services, a long-standing trend that consistently emerges in extant research [73,74,75]. This underreported information may also lead to lower utilization rates of formal treatment services among Latinx than among those in the broader U.S. population. The accumulated NLAAS findings suggest that there might be an alternative reason for this behavior: The specific suffering and needs have not been adequately assessed and/or treated in services designed for mainstream populations. Accordingly, practitioners must move beyond routine psychotherapies to address the pervasive and deleterious impacts of perceived discrimination, stressors induced by acculturation, and other fundamental psychosocial reasons on Latinx behavioral health.

### 4.3. Sociodemographic and Social Participation Factors

Older ages appear to be associated with a greater likelihood of ANX and lower odds of DEP and SUD among Latinx [27,29,45]. Perhaps as a related factor, marriage seems to play a counteracting role against DEP [45]. Concerning the gender effect, Latinas report greater risk for ANX and SUD [47], as well as alcohol use [26]. However, Latinx men had higher rates as current smokers than Latinas [26]. The inconsistent role of these demographic factors may be interpreted in conjunction with different working conditions and related coping strategies in the subgroup Latinx populations.

Within the context of the general U.S. population, it may be an empirical expectation to observe the association between disadvantaged social status, such as unemployment and poverty, and mental health issues, such as DEP among all Latinx [42]. Yet, the detrimental impact of perceived low social status, or perceived downward social mobility, may be more unique to vulnerable Latinx in relation to the large immigration status in this minority population. Low subjective status contributed to both depressive episodes and major depression [31,36,43], as well as AUD [41]. NLAAS researchers have offered a novel insight into the inconsistency between pre-immigration expectations and post-immigration outcomes, such as living conditions, that many Latinx individuals have faced in the United States [31,36]. Unmet dreams after arriving in the U.S. mainland can lead to disappointment and poor psychological and behavioral health. This under-investigated topic could have occurred in other immigrant populations with limited resources for upward social mobility, which deserve more research attention in the future [31].

### 4.4. Protective Factors

Whereas the benefit of social support networks has been a well-established protective factor, the findings on Latinx religious attendance are particularly noteworthy given their acculturation and social status issues [28,44,50]. In most Latinx traditions, faith- and family-oriented traditions inform their community-based beliefs, actions, and behaviors, leading to their collectivist cultural strengths [25,48,65]. This strength differs from mainstream perspectives that prioritize assets like individual strengths, personal autonomy, and achievement. The role of such a culturally based protective factor was found to be particularly true for socially vulnerable Latinx subgroups that were less affluent than for a few of their elite counterparts [76]. Back to the above underutilization issue among Latinx Americans, the secular and individualism-oriented behavioral health services may seek better understanding of this cultural strength so as to effectively communicate and intervene with members from disadvantaged U.S. Latinx communities [77,78].

Nevertheless, the findings have not always been consistent and sometimes even contradictory. For example, in contrast to the protective role of religious attendance and family in other reports, some researchers associated more use of religious coping and perceived greater social support with a high risk of lifetime SUD [27,28]. The author pointed to the likelihood of spurious findings from a cross-sectional survey, because traumatized Latinx individuals could use more religious coping, and the assessment did not distinguish between positive and negative coping styles [28]. The same could be true for social support. Likewise, another report showed the link between low levels of family cohesion with lower risks of SUD, without any interpretation for this hypothesis [46]. Taken together with the above evidence for the risk impacts of misfunctioning family relationships, the mixed findings suggest the role of positive family relationships in Latinx communities may be more complicated. The authors did not provide a satisfactory explanation for another unexpected finding: The coexistence of lower–middle income with lower risks of DEP, compared to middle-class incomes [46]. It is likely that the former group is less acculturated than the latter one, but their stronger sense of community and support system may likely help them cope with the discussed stressors. However, better answers may lie in future prospective studies using advanced statistical modeling to map the true effect in the context of their acculturation and immigration processes [28].

Given the established positive influences in the general population, all these limited findings from the NLAAS should not be a surprise. However, the role of such social relationship-based protective factors should be further examined within the Latinx collectivist culture [29]. Within such culture, members’ personal goals and well-being are, in general, subsumed under those of the collective groups [57,59]. Latinx identity is featured by their extended family-oriented collectivism, as expressed in the cultural phase “mi hijo, mi hija” (“my son, my daughter”) that transcends biological ties and presents a group-based position of their collective relatedness and well-being [79]. This perspective stands in sharp contrast to the assumption of individualistic priority over the collective group in countries espousing mainstream Western tradition [58,80]. Accordingly, behavioral and public health professionals should integrate Latinx cultural views with their personal strengths or resources in order to enhance their mental health and well-being.

It should be noted that studies included in this review are not all rely solely on the NLAAS dataset. Six studies used the National Institute of Mental Health’s Collaborative Psychiatric Epidemiology Studies (CPES) as their data source [30,33,42,45,47,49]. CPES includes three national surveys of mental health in the US population: the National Comorbidity Survey Replication (NCS-R) [81], the National Study of American Life (NSAL) [82], and the National Latino and Asian American Study of Mental Health (NLAAS) [3]. These surveys provided supplementary information for the NLAAS findings, and therefore should not be interpreted as a methodological limitation. The NLAAS is part of the CPES, and all three surveys in the CPES employed the same diagnostic instrument (WMH-CIDI), ensuring consistency in the measurement of ANX, DEP, and SUD. Instead, the use of different data sources provided different analytic perspectives. Studies that only used the NLAAS provided an in-depth examination of how risk and protective factors influence three key outcomes (i.e., ANX, DEP, and SUD) among Latinx populations, with particular attention paid to cultural and immigration-related factors. While studies used the broader CPES sample emphasized cross-ethnic comparisons between Latinxs and their non-Latinx counterparts, both approaches contribute to understanding Latinx populations through within-group and between-group analyses.

### 4.5. Limitations

We should acknowledge the limitations of this systematic review. First, the NLAAS is a 20-year-old database and may not fully represent the current American Latinx population. The demographics and other characteristics of the sample may not entirely align with those of current cohorts and practices. But the core idea concerning positive strengths and mental health remains meaningful for this largest and ever-increasing U.S. minority population. Although this study was conducted many years ago, no other studies have replicated this national representative survey yet. The NLAAS remains unique and informative beyond merely estimating the prevalence of behavioral health conditions. Its continued relevance is evidenced by the fact that the most recent article using this dataset was published in 2024 [35]. To date, this dataset remains the most comprehensive survey on this population, particularly with respect to acculturation-related factors that significantly influence mental health outcomes. The impacts of culturally sensitive factors are unlikely to vanish within a few decades, as the long-standing cross-cultural differences between Northern and Latin America are deeply rooted in diverse traditions. Most importantly, the findings on the detrimental role of discrimination were consistent across the NLAAS publications and are supported by current literature that used other datasets.

Second, to achieve a higher scientific standard, we excluded studies that had not undergone the peer-reviewed process. While the approach strengthens the rigor of our review, it may also have excluded potentially valuable insights from the gray literature, thereby increasing risk of bias. However, the excluded studies were mainly unpublished dissertations or findings only available in conference abstracts without more detailed data, which not only lack the rigorous scholarly review process, but also have insufficient information to be synthetized. For studies included in our review, most were published in high-impact journals after serious review processes. This fact should boost confidence in the overall quality of the included studies.

Third, a major issue of the NLAAS lies in its single-wave design that resulted in cross-sectional studies. The lack of prospective data limits the ability to draw causal conclusions from the available evidence. Also, observational study designs cannot control the impact of unmeasured confounders as effectively as randomized controlled trials can, when examining the relationship between the outcomes and psychosocial determinants. However, we carefully included studies that specifically defined ANX, DEP and/or SUD as the main outcomes, which helped mitigate concerns regarding directional ambiguity. Also, this systematic review has demonstrated the representativeness of the included studies and highlighted the under-investigated factors for cultural strength. As such, our findings may pave the way for future hypotheses to be tested in national surveys and clinical trials, ultimately informing future assessment and intervention efforts.

Finally, although the Latinx population has become more diverse over the past two decades, Cuban, Mexican, and Puerto Ricans still remain the largest subgroups within this racial/ethnic group [2]. However, findings for the overall population of Latinx Americans may not fully reveal the unique chrematistics of each Latinx subgroups, nor those of smaller Latinx subgroups that were collapsed into the “Other” category in the NLAAS. For example, one study showed that though race/ethnic identity might serve as a protective factor for the mental health of all Latinx individuals, Puerto Ricans were the subgroup most likely to use religion as a coping strategy [76]. As “Other” Latinxs have become increasingly represented in the U.S., future investigations should address their distinct challenges, risks, and cultural strengths.

## 5. Conclusions

Our study is the first systematic review to categorize psychosocial risks and protective factors based on three major mental health outcomes (ANX, DEP, and SUD) across the NLAAS studies. The aggregate information provided a comprehensive picture of both generic and unique attributes of the largest minority group in the U.S. Overall, discrimination was the most consistent risk factor across ANX, DEP, and SUD. Family relationships played a key role in ANX and DEP among Latinx individuals whereas acculturation-related factors such as discrimination, acculturative stress, and English proficiency were more salient for SUD. Religious activities appeared to exert protective effects for both DEP and SUD. Gender emerged as an important factor, with female being more likely to experience DEP but less likely to experience SUD than their male counterparts.

To promote culturally sensitive and effective services, several recommendations are proposed below.

First, mental and behavioral health practitioners should integrate the collectivist legacy of Latinx communities as a central focus of treatment rather than relegating it to a peripheral issue or attributing it solely to individual pathology. Behavioral health services for this population should move beyond individual-level therapy to include family and community engagements. For example, given the collectivist traditions of Latinx families and communities, family-centered approaches should be prioritized to reinforce collective bonds and belief systems when family conflict or cohesion emerge as salient. When appropriate, family and group therapy may be recommended, as they may more efficiently address issues related to both family conflicts and cohesion.

Second, based on findings regarding the impact of trauma on the mental health of Latinx individuals, it is essential for providers to implement brief trauma screening. Such assessment should go beyond identifying the negative effects of early mistreatment or negative social relationships in general populations. Instead, providers working with this predominantly immigrant minority group should recognize that acculturative stress and discrimination can increase the risk of ANX, DEP, and SUD. For those who were from different cultural backgrounds, encouraging Latinx clients to share their experiences in this regard may help establish a more trusting therapeutic relationship between the two sides. Such culturally attuned engagement may also address the underutilization of behavioral and mental health service among Latinxs in the U.S.

Third, our review indicates a gap in research on protective factors using the NLAAS dataset, particularly regarding religious involvement and coping mechanisms (e.g., empowerment vs. fatalism). For community-based practices serving Latinx populations, partnerships with faith-based organizations services may be essential. Service providers should also be equipped to explicitly distinguish between positive and negative forms of religious coping, such as empowerment and fatalism.

Fourth, the Latinx population is highly heterogeneous, including multiple national-origin subgroups such as Mexicans, Puerto Ricans, and Cubans. It is essential to recognize that mental and behavioral health risk and protective factors may vary across subgroups and generations. Also, findings from our review suggest that gender influences how Latinx individuals experience ANX, DEP, and SUD. Therefore, gendered experiences warrant further investigation to better understand how immigration, acculturation and trauma experiences interact with gender to shape these outcomes and to inform more tailored interventions.

Lastly, future studies should consider using longitudinal designs to investigate family cohesion, religious activities, and social networks to clarify causal directions, particularly for findings that show mixed risk and protective effects.

## Figures and Tables

**Table 1:** Study characteristics and main findings from included studies.

Author, Year	Population	Sample Size	Age Range (Mean, SD)	Gender	Outcome(s)	Risk Factors	Protective Factors
Ai, Weiss et al., 2014 [29]	Latina Americans	*n* = 1427	18–97 yrs (41.139, 16.08)	100% female	General Anxiety Disorder (GAD), major depressive disorder (MDD).	MDD: U.S.-born. GAD: Age; negative family interactions.	MDD: Education. GAD: Family cohesion
Ai et al., 2016 [27]	Latino American Men	*n* = 1127	18–97 yrs (36.95, 14.24)	100% male	Lifetime SUD	Childhood physical abuse/victimization, age (older), English proficiency, US-born, perceived discrimination.	University graduation
Ai & Lee, 2018 [28]	Latinas Nationwide	*n* = 1427	NR (39.16, 15.74)	100% female	Lifetime SUD	Childhood physical abuse, English proficiency, U.S.-born, perceived discrimination.	Religious attendance
Ai et al., 2024 [35]	Latinx Nationwide	*n* = 2554	18–97 yrs (38.02, 15.03)	48.5% female	Lifetime SUD	Childhood physical mistreatment, English proficiency, U.S.-born, discrimination.	Education, sex
Alcántara et al., 2014 [36]	Latinx Immigrants	*n* = 1561	NR	48.40% female	Major depressive episode (MDE)	Perceived downward social mobility, gender (female), years in the U.S.	Household income
Alcántara, Chen et al., 2015 [37]	Latinx Immigrants	*n* = 1614	NR	55.39% female	MDE	Visits back home.	Remittance burden
Alcántara, Molina et al., 2015 [38]	Latinx Immigrants	*n* = 1629	18–97 yrs (38.61, NR)	55.62% female	Smoking	The number of past-year return visits to the country of origin.	NR
Alegría et al., 2007 [39]	Latinx Subgroups in the U.S.	*n* = 2554	NR	48.50% female	DEP, ANX, SUD	SUD: U.S.-born; English proficiency; second or third generational status.	NR
Canino et al., 2008 [40]	Adult Latinx in the U.S.	*n* = 2546	NR	48.50% female	SUD	Problematic family relations (man), more frequent interaction with friends (woman), more frequent use of English than Spanish.	NR
Cook et al., 2020 [41]	Latinx	*n* = 2554	18–97 yrs (38.1, 0.54)	48.50% female	Alcohol use disorder	U.S.-born, interaction term (U.S. SSS × college degree).	NR
Gavin et al., 2010 [42]	Latinx	*n* = 3258	Male: NR (37.01, 0.58). Female: NR (38.95, 0.51)	56.41% female	MDD	Being out of the labor force.	NR
Jaggers & MacNeil, 2015 [43]	Hispanic Adults Who Immigrated as Youth	*n* = 581	18–97 yrs (35.67, NR)	53.50% female	MDD	Lower SSS, acculturative stress, dissonant acculturation, gender (female).	NR
Kwon et al., 2021 [44]	Latinx Americans	*n* = 2374	NR (38.39, 15.1)	50% female	DEP, ANX	DEP, ANX: Negative life experiences resulting from the 9/11 terrorist attacks, cultural conflict, discrimination.	DEP: Attend religious services.
Lee & Park, 2017 [45]	U.S. Latinx	*n* = 2514	18–97 yrs (37.80, 0.5)	47% female	MDD	Age, sex, and marital status.	NR
Leong et al., 2013 [46]	Latinx American Immigrants	*n* = 2554	18–97 yrs (38.02, 15.03)	48.50% female	DEP, ANX, SUD	DEP, ANX: Acculturative stress. SUD: Low engagement with one’s social network.	DEP, SUD: Family cohesion. ANX, DEP, SUD: Low, hardly ever, or no level of family conflict.
Lo & Cheng, 2012 [30]	Latinx	*n* = 2735	NR	NR	SUD	Sex (male), discrimination, education.	NR
Lo & Cheng, 2018 [47]	Latinx	*n* = 2676	NR (40.46, 15.57)	44.3% male	Mood/anxiety disorders	Gender (female), frequency of discrimination.	NR
Lorenzo-Blanco & Cortina, 2013a [48]	Latinx	*n* = 2554	18–97 yrs (38.02, NR)	48% female	MDD, smoking	MDD: Discrimination, family conflict. Smoking: Discrimination.	NR
Lorenzo-Blanco & Cortina, 2013b [25]	Latinx	*n* = 2554	18–97 yrs (38.02, NR)	48% female	MDD, smoking	MDD, smoking: Discrimination, family conflict.	NR
Molina et al., 2012 [49]	Latinx	*n* = 2554	NR	48.50% female	SUD	NR	Living in neighborhoods with higher concentration of Latinx immigrants.
Molina et al., 2016 [26]	Latinx	*n* = 2554	18–97 yrs (38, 15)	55.87% female	Alcohol and smoke	Alcohol: Discrimination, Smoke: High racial/ethnic identification, gender (female).	Alcohol, smoke: Gender (female).
Moreno & Cardemll, 2018 [50]	Mexican Populations	*n* = 2554	18–88 yrs (36.5, 13.63)	56.50% female	DEP, ANX, SUD	NR	DEP, ANX, SUD: Religious attendance.
Nicklett & Burgard, 2009 [31]	Latinx	*n* = 2554	NR	NR	MDD	A loss of at least 3 steps in SSS, ethnicity, female sex, having resided for a longer than 5 years in the U.S., and being a U.S. citizen.	Gender (male), having resided for less than 5 years in the U.S., and not a U.S. citizen.
Ornelas & Hong, 2012 [51]	Latinx	*n* = 2554	NR	55.87% female	SUD	Unfair treatment, discrimination.	NR
Otiniano et al., 2014 [52]	Latinx	*n* = 2312	18–97 yrs; Latinos: (36.97, 0.63), Latinas (38.47, 0.70)	55.06% female	SUD	Discrimination	NR
Park et al., 2014 [53]	Older Latinx Adults	*n* = 231	71.75–73.86 yrs (72.8, NR)	58.07% female	Late-life DEP	NR	Family cohesion
Polo et al., 2011 [54]	Latinx	*n* = 2554	NR	55.87% female	Social anxiety disorder	Out of labor force, marital status, English proficiency, spoke both English and Spanish while growing up.	Age, foreign-born, Spanish proficiency.
Priest & Denton, 2012 [32]	Latinx	*n* = 2554	NR	NR	ANX	Family discord	Family cohesion
Robinson et al., 2012 [33]	Hispanics	*n* = 3264	NR	NR	ANX	Infrequent religious attendance (less than once per year).	NR
Roth et al., 2022 [55]	Latinx	*n* = 2541	NR (40.61, 15.63)	55.8% female	DEP, ANX, SUD	DEP: Gender (female), previously married, 3rd generation. ANX: Gender (female), 3rd generation. SUD: 2nd or 3rd generation.	ANX, DEP, SUD: Positive acculturative experiences, education. SUD: Gender (female).
Waldman et al., 2021 [34]	Latinx	*n* = 2507	NR	NR	ANX	Post-9/11 discrimination.	NR
Warner et al., 2012 [56]	Hispanic Women in the United States	*n* = 1427	NR	100% female	DEP, ANX	ANX, DEP: Family cultural conflict. DEP: Witness violence. ANX: Witness violence, beaten, sexual assault.	NR

Note. NR: Not reported.

## Data Availability

No new data were created or analyzed in this study. Data sharing is not applicable to this article.
